# Lack of Skeletal Muscle IL-6 Affects Pyruvate Dehydrogenase Activity at Rest and during Prolonged Exercise

**DOI:** 10.1371/journal.pone.0156460

**Published:** 2016-06-21

**Authors:** Anders Gudiksen, Camilla Lindgren Schwartz, Lærke Bertholdt, Ella Joensen, Jakob G. Knudsen, Henriette Pilegaard

**Affiliations:** Section for cell biology and physiology, Department of Biology, University of Copenhagen, Copenhagen, Denmark; University of Birmingham, UNITED KINGDOM

## Abstract

Pyruvate dehydrogenase (PDH) plays a key role in the regulation of skeletal muscle substrate utilization. IL-6 is produced in skeletal muscle during exercise in a duration dependent manner and has been reported to increase whole body fatty acid oxidation, muscle glucose uptake and decrease PDHa activity in skeletal muscle of fed mice. The aim of the present study was to examine whether muscle IL-6 contributes to exercise-induced PDH regulation in skeletal muscle. Skeletal muscle-specific IL-6 knockout (IL-6 MKO) mice and floxed littermate controls (control) completed a single bout of treadmill exercise for 10, 60 or 120 min, with rested mice of each genotype serving as basal controls. The respiratory exchange ratio (RER) was overall higher (P<0.05) in IL-6 MKO than control mice during the 120 min of treadmill exercise, while RER decreased during exercise independent of genotype. AMPK and ACC phosphorylation also increased with exercise independent of genotype. PDHa activity was in control mice higher (P<0.05) at 10 and 60 min of exercise than at rest but remained unchanged in IL-6 MKO mice. In addition, PDHa activity was higher (P<0.05) in IL-6 MKO than control mice at rest and 60 min of exercise. Neither PDH phosphorylation nor acetylation could explain the genotype differences in PDHa activity. Together, this provides evidence that skeletal muscle IL-6 contributes to the regulation of PDH at rest and during prolonged exercise and suggests that muscle IL-6 normally dampens carbohydrate utilization during prolonged exercise via effects on PDH.

## Introduction

Skeletal muscle possesses a remarkable ability to regulate substrate use with changing substrate availability and energy demands [1,2]. As the Randle cycle originally proposed [3], lipids and carbohydrates (CHO) play competitive but equally essential roles as substrate in energy production in muscle. The coordinated dynamic switch between these substrates is vital to sustaining ATP production during prolonged metabolic challenges such as exercise. The demand for energy supply increases many fold over resting state requirements at the onset of exercise and simultaneous induction of numerous metabolic pathways are initiated across tissues in order to increase both fat and carbohydrate availability and oxidation [4,5]. During prolonged low to moderate intensity exercise, a reciprocal shift from CHO to lipid oxidation occurs in skeletal muscle in order to spare muscle glycogen stores and hence prolong the ability for the muscle to contract [6,7]. However, the molecular mechanisms behind this remain to be elucidated.

The pyruvate dehydrogenase complex (PDC) represents the only point of entry for CHO derived fuel into the mitochondria for complete oxidation [8,9] and is therefore seen as a metabolic gatekeeper. Located within the mitochondrial matrix, the PDC exerts its role by catalyzing the rate-limiting and irreversible decarboxylation of pyruvate thereby connecting glycolysis with the Krebs cycle. The PDC is composed of multiple copies of the three enzymatic subunits E1, E2, and E3, where the tetrameric (2α/2β) E1 enzyme, also termed pyruvate dehydrogenase (PDH), is the initial catalyst in the decarboxylation step (Harris, 2001). Covalent modifications by means of phosphorylation of at least four different serine sites (site 1: Ser293; site 2: Ser300; site 3: Ser232, and site 4: Ser295) on the E1 enzyme have so far been thought to be the main regulatory mechanism controlling the activity of the PDC, although allosteric regulation by the substrates, pyruvate and NAD^+^, and the products, acetyl-CoA and NADH, as positive and negative allosteric effectors, respectively, may also contribute [10–12]. The activity of PDH in its active form (PDHa activity) is inhibited by phosphorylation catalyzed by 4 isoforms of PDH kinases (PDK) and stimulated by dephophorylation catalyzed by 2 isoforms of PDH phosphatases (PDP), of which PDK2 and PDK4 and the Ca^2+^-sensitive PDP1 have been suggested to be the most highly expressed isoforms in skeletal muscle [13,14]. PDHa activity is rapidly increased within the first minutes of exercise strongly correlated with exercise intensity [15–17]. In addition, PDHa activity has been shown to decrease after 2h of exercise in humans [12,18] reflecting a dominant reliance on CHO at the onset of exercise which gradually decreases over time as FFA available and lipid oxidation increase [7,18,19]. Furthermore, the exercise-induced regulation of PDHa activity has been shown to be associated with opposite changes in PDH phosphorylation in human skeletal muscle [19–21] indicating phosphorylation as an important regulatory mechanism in the regulation of PDH. Moreover, recent studies have provided evidence for acetylation of PDH-E1α, with the NAD^+^-dependent deacetylase sirtuin 3 (SIRT3) shown to target PDH-E1α, possibly playing an important role in maintaining the tight control of the complex [22,23].

Although the regulation of PDHa activity through post-translational modifications is well established, the signaling pathways inducing these modifications remain to be fully investigated. Previous studies suggest that interleukin (IL) 6 may play a role. Thus, human studies have shown that IL-6 is produced in and released from skeletal muscle during exercise in a duration and intensity dependent manner [24,25]. Furthermore, IL-6 infusion in humans has been shown to increase skeletal muscle fat oxidation [26], and some studies link IL-6 to augmented glucose uptake during exercise in humans [27,28], although others report no correlation between plasma IL-6 concentrations and glucose uptake in human [29] and mouse muscle [30]. In addition, injections with physiologically relevant doses of recombinant IL-6 have been shown to decrease PDHa activity in mouse skeletal muscle during fed conditions [31] and rodent studies have indicated that IL-6 activates AMP activated protein kinase (AMPK) [27,32]. Moreover, because lack of AMPKα2 has been shown to enhance PDHa activity in mouse skeletal muscle [33], a regulatory metabolic axis may exist between IL-6, AMPK, and PDH during exercise. Taken together, IL-6 seems to be a potential candidate for regulating skeletal muscle PDH during prolonged exercise.

The purpose of this present study was to test the hypothesis that skeletal muscle IL-6 contributes to the regulation of PDH in mouse skeletal muscle at rest as well as during prolonged exercise and that this is associated with IL-6 mediated regulation of AMPK.

## Methods

### Animals

Male C57BL/6 mice carrying loxP inserts that flanked exon 2 of the IL-6 gene as previously described [34] were crossbred with C57BL/6 mice carrying the cre recombinase gene under control of the myogenin promoter generating mice carrying the loxP inserts (floxed), serving as control mice, and skeletal muscle-specific IL-6 knockouts (IL-6 MKO) as previously shown [35]. Animals were maintained on a 12:12 light-dark cycle at 22°C with ad libitum access to water and chow diet (Altromin 1314F, Brogaarden, Lynge, Denmark) and were therefore in a fed state before the exercise intervention. All experiments were approved by the Danish Animal Experimental expectorate and complied to the European Convention for the protection of vertebrate animals used for experiments and other scientific purposes (Council of Europe no. 123. Strasbourg, France 1985).

### Endurance test protocol

Mice also included in another study (Knudsen et al., 2015) were used to estimate relative endurance capacity. Mice performed a graded treadmill test (TSE systems, Bad Hamburg, Germany) at 10 degree incline and an initial speed of 11 m/min for 2 min. This was followed by gradually increasing the velocity every 12^th^ min to 14, 16, 18, 20, and 22 m/min, after which the incline was increased by 2 degrees every 12^th^ min until exhaustion. Exhaustion was characterized as the point where mice could not be encouraged to run further despite being subjected to gentle electrical shock and stimulation with compressed air.

### Acute exercise procedure

At 3 months of age, mice were housed individually and acclimatized to treadmill running (TSE Systems, Bad Hamburg, Germany) 10 min, 2 times a day for 5 days with 1 day of rest prior to the experimental day.

The respiratory exchange ratio (RER) was determined during running (n = 5–7) on treadmills connected to a Phenomaster unit (TSE Systems, Bad Hamburg, Germany) at 14 m/min with a 10° incline for 120 min.

On the day of the main experiment, mice were prompted to run at 14 m/min with a 10° incline (n = 10) for 10 min, 60 min, or 120 min between 8:00 and 10:30 AM before being euthanized by cervical dislocation. Quadriceps muscles were swiftly removed and snap frozen in liquid nitrogen. Trunk blood was obtained in EDTA containing tubes and plasma was obtained after centrifugation. Both muscle and plasma were stored at -80°C.

### Plasma analyses

Plasma NEFA concentrations were measured colorimetrically using a NEFA-HR (2) kit according to the manufacturer´s guidelines (WAKO Diagnostics GmbH, Germany). Plasma IL-6 was measured using a mesoscale v-plex kit according to the manufacturer’s guidelines (MSD, Rockville, MD, USA). Plasma glucose and lactate were measured fluorometrically as previously described [36].

### Muscle analyses

#### Muscle glucose, lactate, glucose-6 phosphate and glycogen

Whole quadriceps muscles were crushed in liquid nitrogen to achieve tissue homogeneity. For measurements of muscle glucose, lactate, and glucose-6-phosphate (G-6-P) 10-15mg of crushed muscle tissue was extracted in perchloric acid (PCA) and neutralized to a pH of 7–8.

Muscle glycogen was determined fluorometrically as glycosyl units after hydrolyzing 10–15 mg wet weight muscle samples by boiling for 2h in HCl (1M) as previously described [36].

### HAD and CS activity

The maximal activity of citrate synthase (CS) was measured using a citrate synthase Assay Kit (Sigma-Aldrich) according to manufacturer’s guidelines and maximal β-hydroxyacyl-CoA dehydrogenase (HAD) activity was measured as previously described [37].

### Immunoblotting

Crushed muscle samples (25-35g) were homogenized in lysis buffer (10% glycerol, 20 mM Na-pyrophosphate, 150 mM NaCl, 50 mM HEPES, 1% NP-40, 20 mM β-glycerophosphate, 10 mM NaF, 1 mM EDTA, 1 mM EGTA, 20 μg/ml aprotinin, 10 μg/ml leupeptin, 2 mM Na_3_VO_4_, 3 mM benzamidine, pH 7.5) using a Tissue Lyser II (Qiagen, Germany). Samples for immunoprecipitation had deacetylase inhibitors (nicotinamide (1mM) and sodium butyrate (5mM)) added to the lysis buffer. Protein content in each of the samples was determined using the bicinchoninic acid method (Thermo Fischer Scientific, USA) and content was adjusted with sample buffer to a concentration of 2μg/μl for STAT3^Tyr705^ and 1μg/μl for the remaining proteins. Protein phosphorylation and protein levels were determined by SDS-PAGE using hand casted gels and western blotting. Membranes were incubated in primary antibody for determination of AMPKα2, PDK4 and PDH-E1α protein, PDH-E1α^Ser293^, PDH-E1α^Ser300^, and PDH-E1α^Ser295^phosphorylation (all kindly provided by Professor Graham Hardie, University of Dundee, Scotland), P38 protein, P38^Thr180/Tyr182^ phosphorylation, Hexokinase (HK) II protein, AMPK^Thr172^phosphorylation, signal transducer and activator of transcription (STAT3) protein, STAT3^Tyr705^ phosphorylation (#9212, #4511, #2867, #2535, #9139, #9138, and #9441, respectively, Cell Signaling Technologies, Danvers, MA, USA), acetyl CoA carboxylase (ACC)^Ser212^ phosphorylation and PDH-E1α^Ser232^ phosphorylation (07–303 and #AP1063, respectively, EMD Millipore, Bedford, USA), PDK1 and OXPHOS proteins (ab90444 and ab110413, respectively, Abcam, Cambridge, U.K.), PDK2 protein (ST1643, CalBioChem, Bedford, USA), PDP1 protein (Sigma-Aldrich, St. Louis, USA) and GLUT4 protein (PAI-1065, ABR, Connecticut, USA). Species-specific horseradish peroxidase conjugated immunoglobulin secondary antibodies (DAKO, Denmark) were used for incubation the following day (ACC2 was incubated with streptavidin as primary antibody) and protein bands were subsequently visualized using an ImageQuant LAS 4000 imaging system and quantified with ImageQuant TL 8.1 software (GE Healthcare, Freiburg, Germany).

### Immunoprecipitation for protein acetylation

A total of 100 μg of protein from lysate was immunoprecipitated for the determination of PDH acetylation state. Briefly the lysate was added to washed protein G agarose beads (EMD Millipore, Bedford, USA) in a 50:50 solution with PBS buffer containing 10% Triton X with 2μg of PDH-E1α antibody. The samples were rotated end over end at 4°C overnight and on the subsequent day the beads were washed, sample buffer was added and the samples were shortly heated to 96°C for 3 minutes. The beads were spun down and lysate loaded on a hand-casted gel for SDS-page and western blotting as described above and incubated with total lysine acetylation antibody (#9441, Cell Signaling Technologies, Danvers, MA, USA). Acetylated protein was normalized to the amount of precipitated PDH-E1α protein content for each sample.

### RNA isolation, reverse transcription, and Real-time PCR

RNA was isolated using a modified guanidium-thiocyanate-chloroform protocol as previously described [38–40]. Reverse transcription was performed on 3μg of RNA from quadriceps muscle using the Superscript II RNAse H^-^ system and oligodT (Life Technologies, Nærum, Denmark). To determine mRNA content, real-time PCR was performed using the ABI-7900 Sequence Detection System (Applied biosystems, Foster City, CA, USA). A fragment of cDNA was amplified using the following oligo sequences for IL-6: forward 5´GCTTAATTACACATGTTCTCTGGGAAA3´, reverse 5´CAAGTGCATCGTTGTTCATAC3´ and Taqman probe 5`ATCAGAATTGCCATTGCACAACTCTTTTCTCAT3´, and for SOCS3: forward 5´GCCACCTGGACTCCTATGAGAA3´, reverse 5´GAGCATCATACTGATCCAGGAACTC3´, and Taqman probe 5´TGACCCAGCTGCCTGGAC CCATT3´. Samples were run in triplicates together with a serial dilution made from a pool of aforementioned samples. The serial dilution was used to create a standard curve for quantification of the specific amount of mRNA from obtained sample Ct values. For each sample, the target mRNA content was normalized to β-actin mRNA, which was not affected by either exercise or genotype.

### PDHa activity

PDHa activity (activity of PDH in the active form) was determined after homogenizing 10-15mg of wet weight muscle tissue and snap-freezing the homogenate in liquid nitrogen as previously described [15,19,41,42] and normalized to creatine content in each muscle sample.

### Statistics

All values are expressed as means ± SE. A two-way ANOVA was used to test the effects of genotype and exercise. When a main effect was detected a Student-Newmann-Keuls test was applied as a post-hoc test to locate differences. For single group data, a student’s t-test was used to test if a difference was present. Significance was accepted at P<0.05 and a tendency for 0.05≤P≤0.1. Results were analyzed using Sigmaplot 13.0 (Systat, USA).

## Results

### Endurance Test

**Fig 1:**
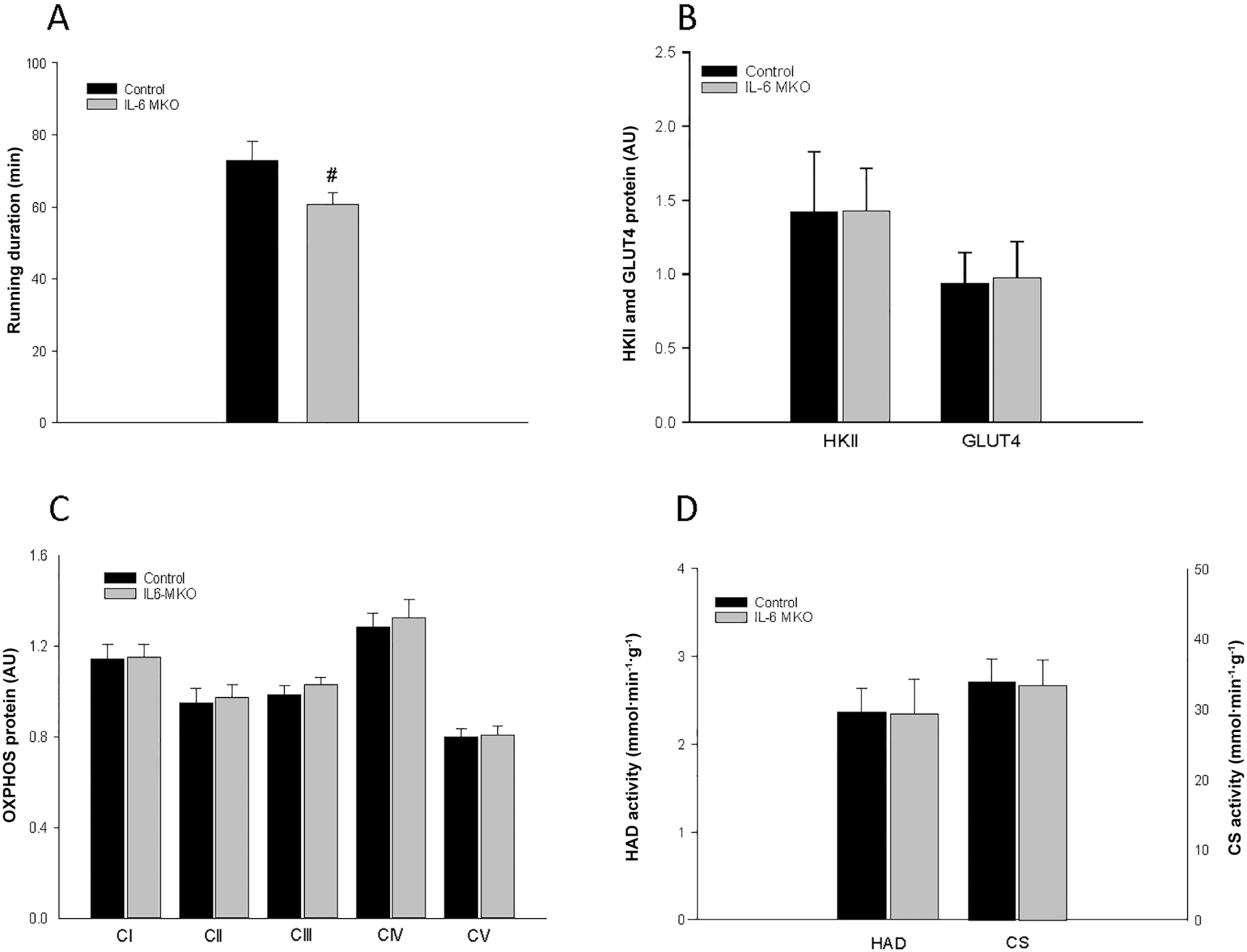
**A** Running duration (min) in a graded treadmill endurance test and basal skeletal muscle **B)** HKII and GLUT4 protein content, **C)** OXPHOS complex (I-V) protein content and **D)** HAD and CS activity in skeletal muscle specific IL-6 knockout (IL-6 MKO) and littermate floxed control (Control) mice. Protein levels are given in arbitrary units (AU). Values are given as mean ± SE; n = 6–10 in A and n = 9–10 in B-D. #: significantly different from control, P<0.05;

Running duration was shorter (P<0.05) for IL-6 MKO mice than for control mice (Fig 1A).

#### HK II, GLUT 4, OXPHOS complexes, HAD and CS activity

There were no differences in basal protein content of HKII and GLUT4 (Fig 1B) or OXPHOS complexes in skeletal muscle of control and IL-6 MKO mice (Fig 1C). Similarly, there were no genotype differences in basal CS or HAD activity in skeletal muscle (Fig 1D).

### Plasma IL-6

**Fig 2:**
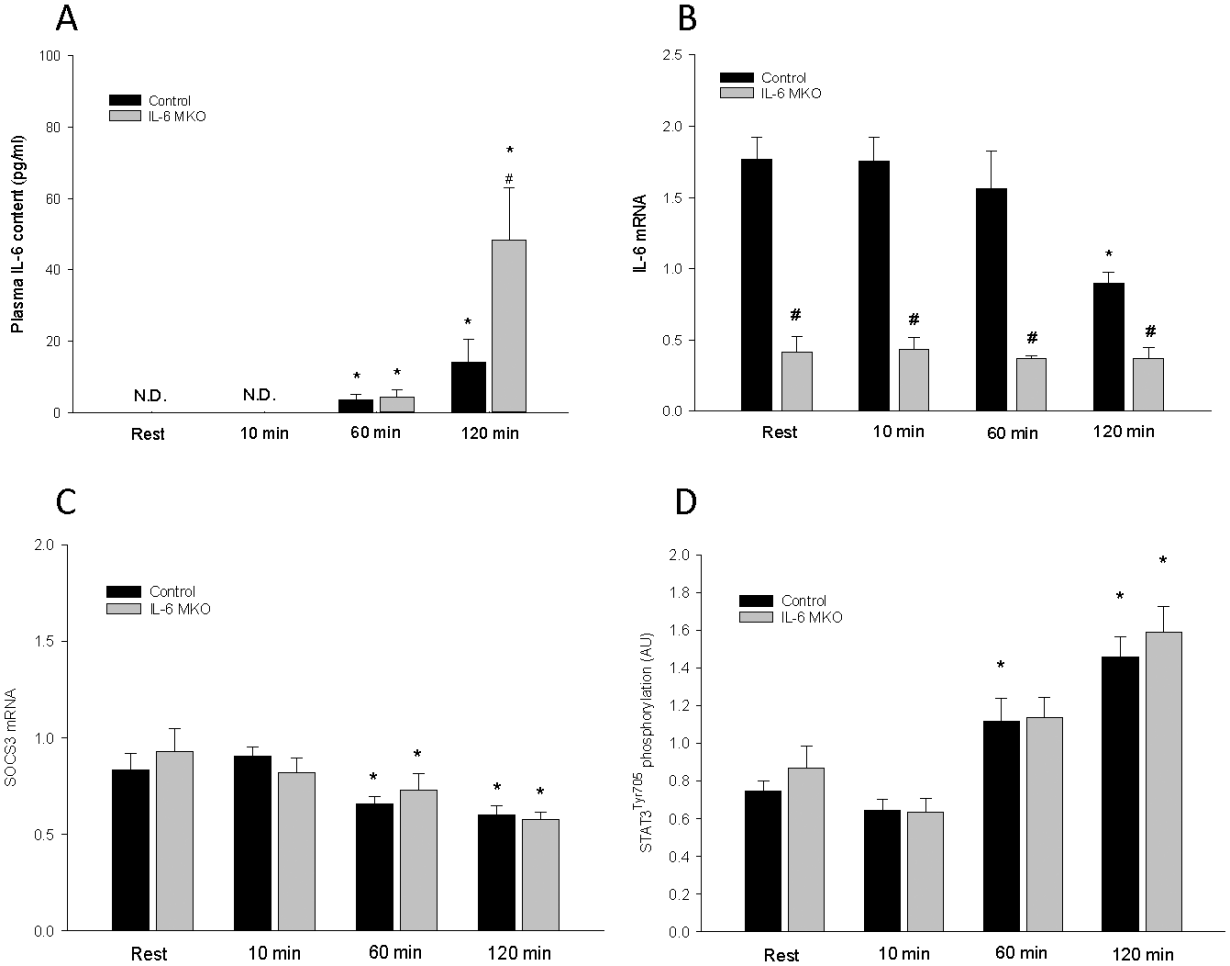
**A)** Plasma IL-6 and skeletal muscle **B)** IL-6 mRNA, **C)** SOCS3 mRNA and **D)** STAT3 Tyr705 phosphorylation in skeletal muscle specific IL-6 knockout (IL-6 MKO) and littermate floxed control (Control) mice at rest and after 10, 60 or 120 min of exercise. Protein levels are given in arbitrary units (AU). Values are given as mean ± SE; n = 10. *: significantly different from rest within given genotype, P<0.05. #: significantly different from control within given time point, P<0.05.

Plasma IL-6 was non-detectable at rest and at 10 min of exercise in both control and IL-6 MKO mice, but increased (P<0.05) in both genotypes after 60 min and 120 min of exercise. IL-6 MKO had higher (P<0.05) plasma IL-6 than control mice after 120 min of exercise (Fig 2A).

#### IL-6 and SOCS3 mRNA

There was an overall difference (P<0.05) in IL-6 mRNA content between control and IL-6 MKO mice with ~300% higher (P<0.05) IL-6 mRNA content in control than IL-6 MKO mice at rest and after 10 min and 60 min of exercise, and ~140% higher (P<0.05) in control than IL-6 MKO mice after 120 min of exercise (Fig 2B). There was no effect of exercise on muscle IL-6 mRNA at 10 min and 60 min of exercise, while IL-6 mRNA content was lower (P<0.05) at 120 min of exercise than rest in control mice. The residual IL-6 mRNA measured in the IL-6 MKO mice is assumed to originate from non-muscle tissue. SOCS3 mRNA was lower (P<0.05) at 60 min and 120 min of exercise than at rest in both genotypes with no difference between genotypes at any of the time points (Fig 2C).

#### STAT3

STAT3^Tyr705^phosphorylation in muscle was higher (P<0.05) than at rest after 60 min of exercise in control mice and after 120 min in both genotypes (Fig 2D). There was no difference in STAT3^Tyr705^phosphorylation and STAT3 protein between the genotypes.

#### Plasma glucose, NEFA, and lactate

The plasma glucose concentration was higher (P<0.05) in IL-6 MKO than in control mice at rest. The plasma glucose level was higher (P<0.05) at 10 min and 60 min of exercise than at rest in control mice, whereas plasma glucose was lower (P<0.05) after 120 min of exercise than at rest in IL-6 MKO mice (Table 1). While plasma NEFA concentrations did not change significantly during exercise in control mice, the plasma NEFA level in IL-6 MKO mice was lower (P<0.05) after 10 and 60 min of exercise and higher (P<0.05) after 120 min of exercise than at rest. The plasma NEFA was lower (P<0.05) in IL-6 MKO than control mice at 10 min of exercise and tended to be higher (0.05≤P<0.1) in IL-6 MKO than control mice at 120 min of exercise (Table 1). The plasma lactate concentration was lower (P<0.05) after 120 min of exercise than at rest in both genotypes. Furthermore, the plasma lactate concentration was lower (P<0.05) in IL-6 MKO than in control mice at rest and after 10 min of exercise (Table 1).

**Table 1:** Plasma concentrations of glucose, NEFA, and lactate. Plasma glucose, non-esterified fatty acids (NEFA), and lactate in skeletal muscle specific IL-6 knockout (IL-6 MKO) and littermate floxed controls (Control) mice at rest and after 10, 60 or 120 min of exercise. Values are given as mean ± SE; n = 9–10.

	Rest		10 min		60 min		120 min	
	Control	IL6-MKO	Control	IL6-MKO	Control	IL6-MKO	Control	IL6-MKO
**Glucose (mmol/L)**	**7.29 ±0.25**	**8.26 ±0.31**^#^	8.45 ±0.20*	**8.38 ±0.42**	8.79 ±0.35*	**8.97 ±0.20**	**7.56 ±0.53**	6.89 ±0.46*
**NEFA (mmol/L)**	**0.86 ±0.09**	**0.76 ±0.05**	**0.75 ±0.05**	0.57 ±0.03*^,^^#^	**0.68 ±0.05**	0.65 ±0.03*	**0.85 ±0.04**	0.99 ±0.03*^,^^(^^#^^)^
**Lactate (mmol/L)**	**6.76 ±0.68**	**4.64 ±0.36**^#^	**6.70 ±0.70**	**4.21 ±0.29**^#^	**5.18 ±0.75**	**4.50 ±0.63**	4.16 ±0.67*	2.44 ±0.33*

*: significantly different from rest within given genotype, P<0.05.

^#^: significantly different from control within given time point, P<0.05. (*): Tendency to be significantly different from rest within given genotype, 0.05<P<0.01, (#): Tendency to be significantly different from control within given time point, P<0.05.

### Muscle glucose, G-6-P, glycogen, lactate and acetyl CoA

Muscle glucose concentration in control mice was higher (P<0.05) after 10 min and tended to be higher (0.05≤P<0.1) after 60 min of treadmill exercise than at rest. Furthermore, muscle glucose was higher (P<0.05) in control than in IL-6 MKO mice at both these time points (Table 2). Muscle G-6-P tended to be lower (0.05≤P<0.1) after 10 and 60 min of exercise than at rest in both genotypes. G-6-P in IL-6 MKO mice was lower (P<0.05) after 60 min than at rest and in both genotypes lower (P<0.05) after 120 minutes of exercise than at rest (Table 2). Muscle glycogen gradually decreased with exercise (P<0.05) and was different from rest at every time point without any significant differences between control and IL-6 mice (Table 2). It may be noted that the difference in muscle glycogen from Rest to 120 min of exercise was 50% less in IL-6 MKO than Control mice, but this difference was not significant. Furthermore, the muscle lactate concentration was lower (P<0.05) after 120 min of exercise than at rest in both genotypes (Table 2). Muscle acetyl CoA was not affected by exercise or genotype.

**Table 2:** Skeletal muscle glucose, G-6-P, glycogen, lactate, and acetyl CoA content. Skeletal muscle glucose, glucose -6 phosphate (G-6-P), glycogen, lactate and acetyl CoA in skeletal muscle specific IL-6 knockout (IL-6 MKO) and littermate floxed controls (Control) mice at rest and after 10, 60 or 120 min of exercise. Values are given as mean ± SE; n = 9–10.

	Rest		10 min		60 min		120 min	
	Control	IL6-MKO	Control	IL6-MKO	Control	IL6-MKO	Control	IL6-MKO
**Glucose (mmol/kg)**	**0.41 ±0.04**	**0.2 ±0.04**	0.66 ±0.08*	**0.46 ±0.05**^#^	**0.62 ±0.08**(*)	**0.41 ±0.05**^#^	**0.38 ±0.03**	**0.31 ±0.05**
**G-6-P (mmol/kg)**	**5.85 ±0.14**	**5.86 ±0.19**	**5.64 ±0.21**	**5.39 ±0.16**(*)	**5.31 ±0.11**(*)	5.14 ±0.14*	5.08 ±0.18*	4.86 ±0.21*
**Glycogen (mmol/kg)**	**16.25 ±1.1**	**18.41 ±1.49**	12.14 ±0.39*	12.35 ±1.14*	7.87 ±0.84*	10.64 ±0.78*	8.60 ±1.17*	6.93 ±0.78*
**Lactate (mmol/kg)**	**3.83 ±0.29**	**4.37 ±0.54**	**3.97 ±0.55**	**3.88 ±0.19**	**3.46 ±0.51**	**3.49 ±0.39**	2.85 ±0.48*	2.72 ±0.74*
**Acetyl CoA (mmol/kg)**	**0.952±0.06**	**0.851±0.05**	**0.821±0.03**	**0.865±0.04**	**0.831±0.03**	**0.860±0.03**	**0.905±0.03**	**0.798±0.03**

*: significantly different from rest within given genotype, P<0.05.

^#^: significantly different from control within given time point, P<0.05. (*): Tendency to be significantly different from rest within given genotype, 0.05<P<0.01. (#): Tendency to be significantly different from control within given time point, P<0.05.

### AMPK and ACC

**Fig 3:**
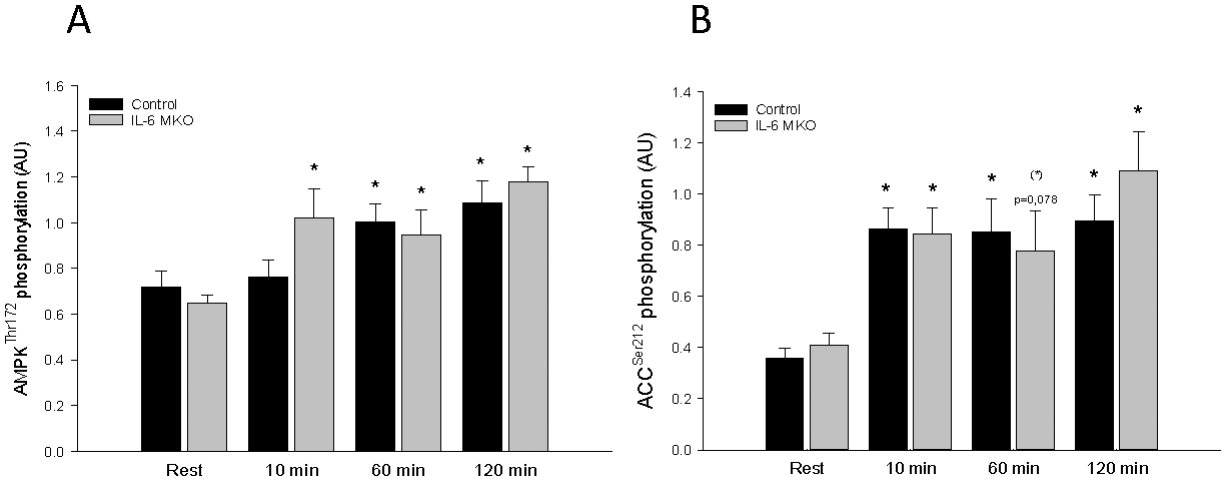
**A)** AMP-activated protein kinase (AMPK) Thr172 phosphorylation and **B)** Acetyl-CoA carboxylase 2 (ACC2) phosphorylation in skeletal muscle from skeletal muscle specific IL-6 knockout (IL-6 MKO) and littermate floxed controls (Control) mice at rest and after 10, 60 or 120 min of exercise. Values are given as mean ± SE; n = 9–10. Phosphorylation levels are given in arbitrary units (AU). *: significantly different from rest within given genotype, P<0.05. #: significantly different from control within given time point, P<0.05. (*): Tendency to be significantly different from rest within given genotype, 0.05<P<0.01.

AMPK^Thr172^phosphorylation in IL-6 MKO mice was higher (P<0.05) after 10, 60, and 120 min of exercise than at rest and in control mice higher (P<0.05) after 60 and 120 min than at rest (Fig 3A). There were no genotype differences in skeletal muscle AMPK^Thr172^phosphorylation and AMPKα2 protein (Fig 3A). ACC^Ser212^phosphorylation after 10, 60, and 120 min of exercise was higher (P<0.05), apart from MKO mice having a tendency to be higher (0.5≤ P ≤0.1) at 60 min (P = 0.078), than at rest in both genotypes (Fig 3B). There were no genotype differences in skeletal muscle ACC^Ser212^phosphorylation and ACC2 protein (Fig 3B).

### PDH-E1α phosphorylation, PDK and PDP protein

**Fig 4:**
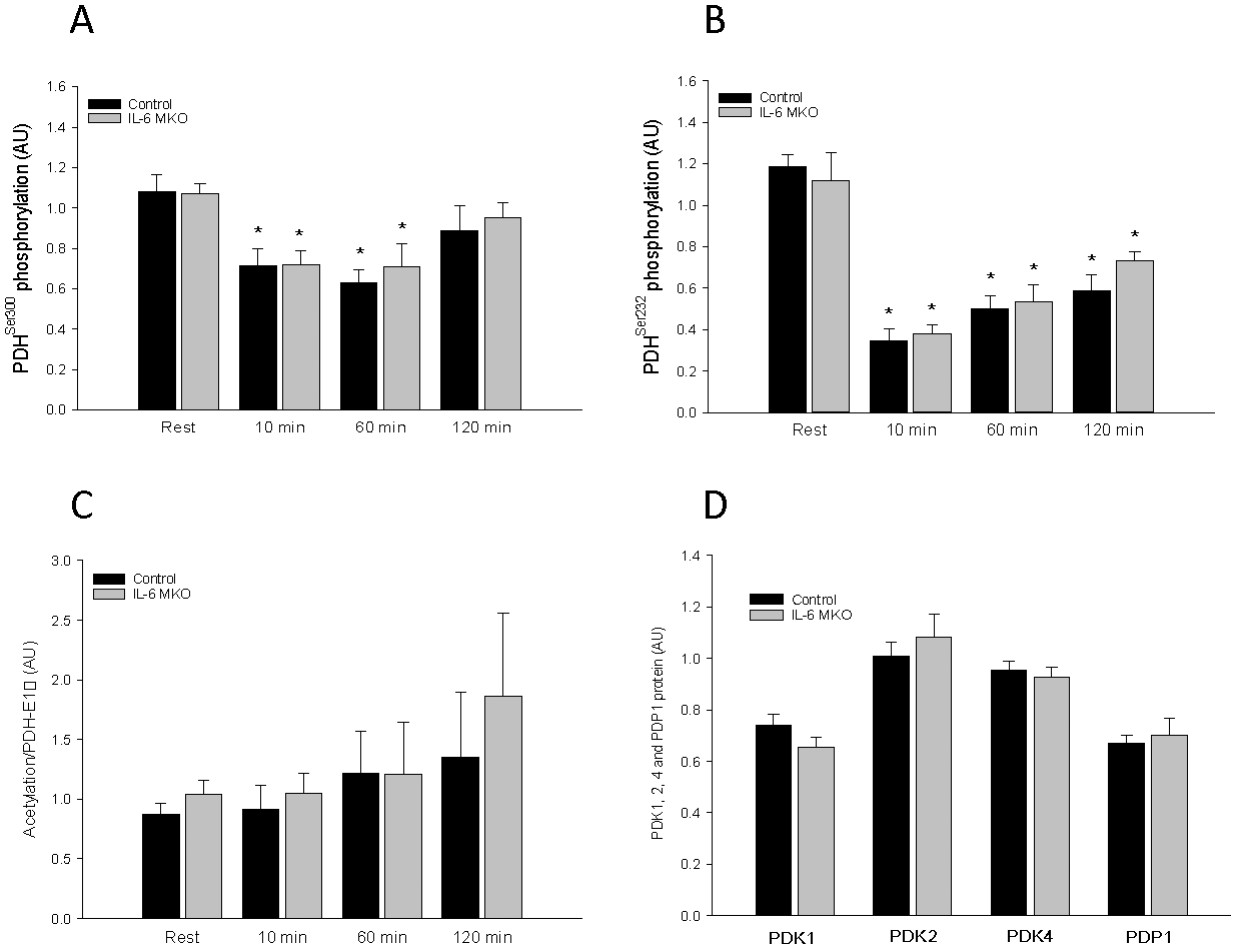
**A)** PDH Ser300 phosphorylation, B**)** PDH Ser232 phosphorylation, C**)** PDH acetylation in skeletal muscle from skeletal muscle specific IL-6 knockout (IL-6 MKO) and littermate floxed controls (Control) at rest and after 10, 60 or 120 min of exercise and D) basal PDK1, PDK2, PDK4 and PDP1. Values are given as mean ± SE; n = 10 in A,B and D and n = 5 in C. *: significantly different from rest within given genotype, P<0.05. #: significantly different from control within given time point, P<0.05.

While skeletal muscle PDH-E1α^Ser293^ and PDH-E1α^Ser295^ phosphorylation (site 1 and 4) did not change during the 120 min of treadmill exercise, PDH-E1α^Ser300^ and PDH-E1α^Ser232^phosphorylation (site 2 and 3) was lower (P<0.05) after 10 and 60 min of exercise than at rest in both control and IL-MKO mice (Fig 4A and 4B). There were no differences in skeletal muscle PDK1, PDK2, PDK4 or PDP1 protein content between time points or genotypes (Fig 4D).

### PDH acetylation and SIRT3

There were no differences in skeletal muscle SIRT3 or PDH-E1α acetylation (Fig 4C).

### PDHa activity

**Fig 5:**
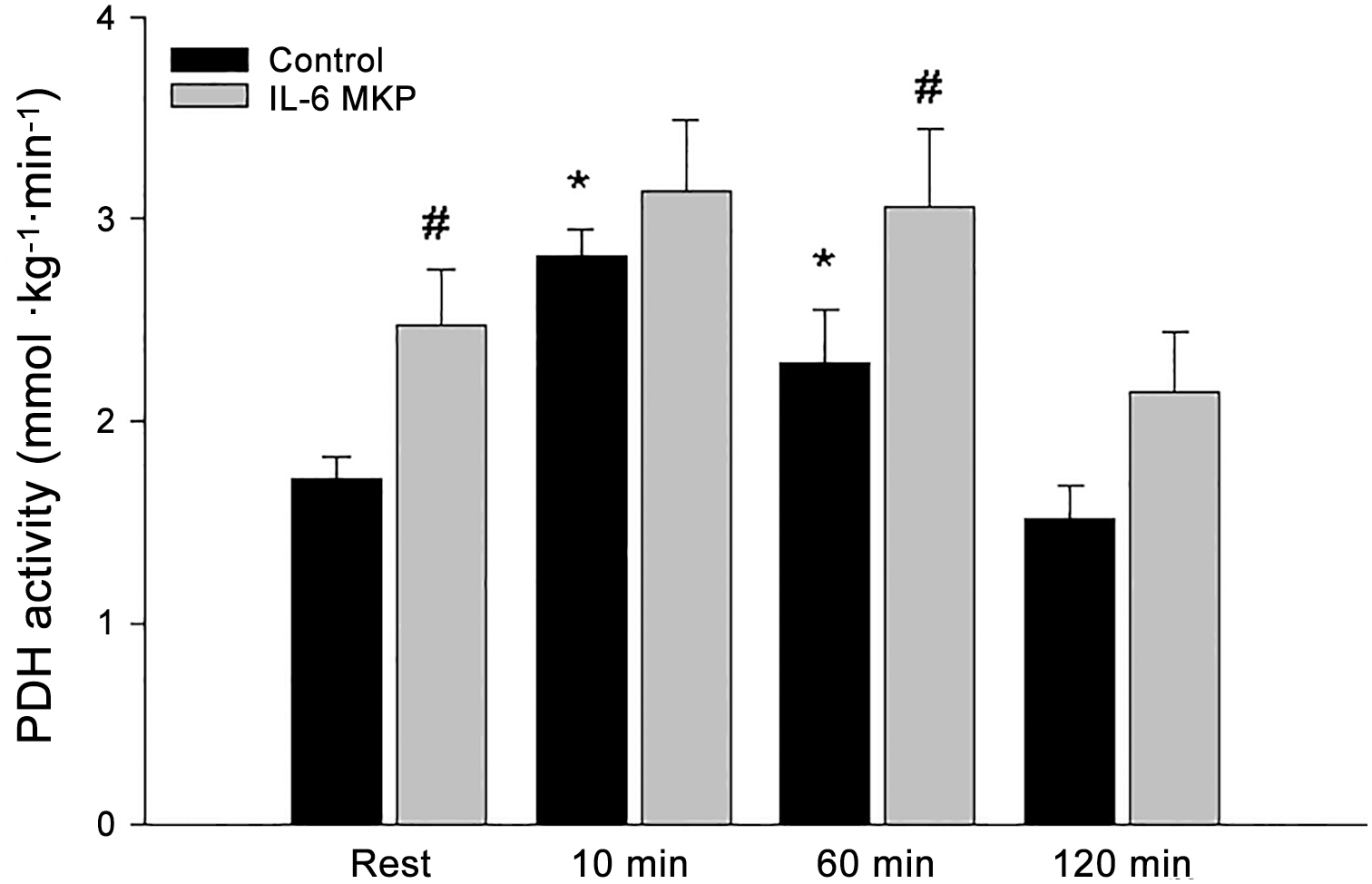
PDHa activity in skeletal muscle from skeletal muscle specific IL-6 knockout (IL-6 MKO) and littermate floxed controls (Control) at rest and after 10, 60 or 120 min of exercise. Values are given as mean ± SE; n = 10. *: significantly different from rest within given genotype, P<0.05. #: significantly different from control within given time point, P<0.05.

PDHa activity was higher (P<0.05) after 10 and 60 min of exercise than at rest in control mice, while there was no effect of exercise on PDHa activity in IL-6 MKO mice. The PDHa activity was higher (P<0.05) in IL-6 MKO than control mice at rest and 60 min of exercise (Fig 5). There were no differences in skeletal muscle PDH-E1α protein.

### Indirect calorimetry

**Fig 6:**
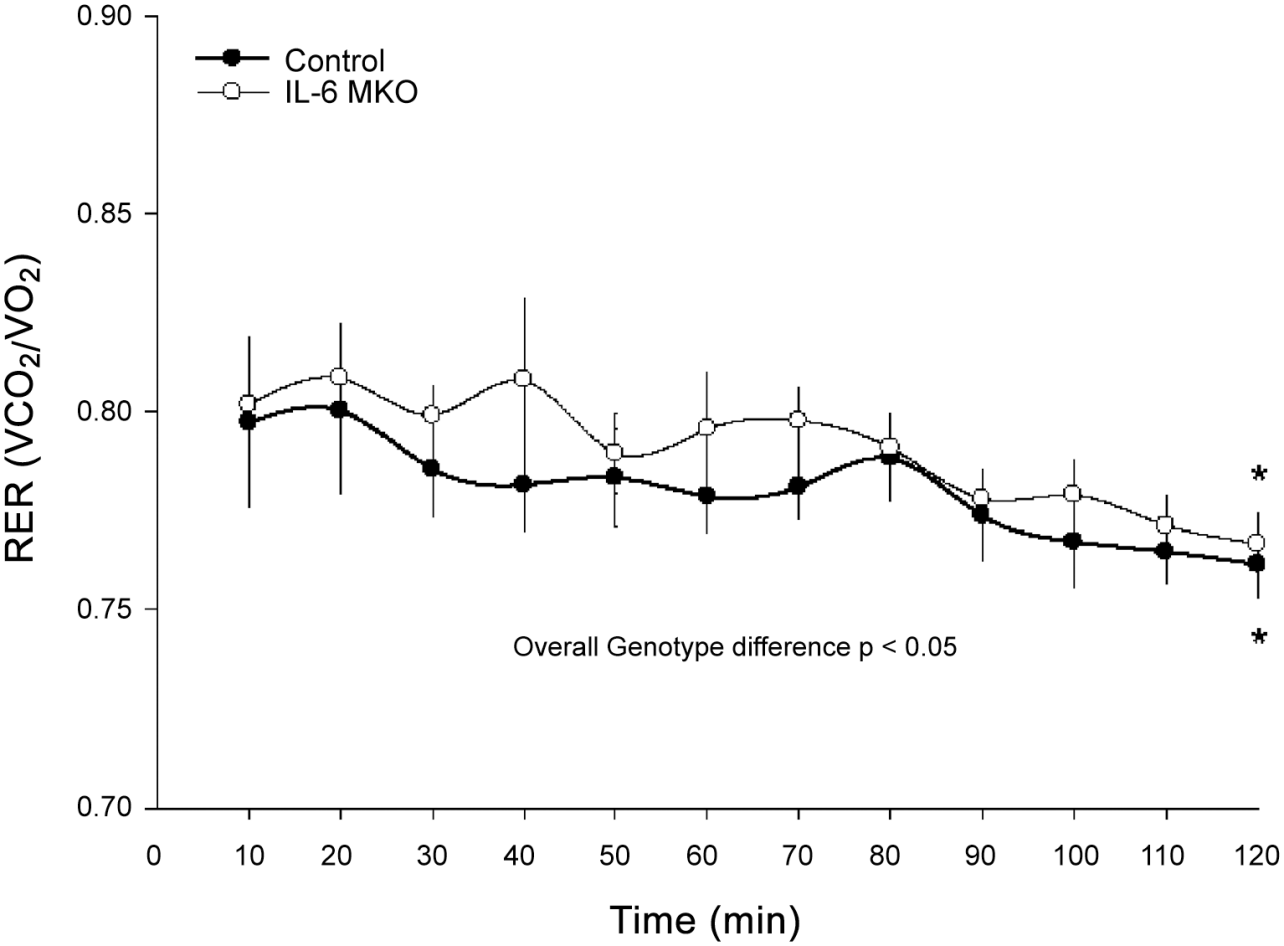
Respiratory Exchange Ratio (RER) in skeletal muscle specific IL-6 knockout (IL-6 MKO) and littermate floxed controls (Control) during 120 min of metabolic treadmill exercise. Values are given as mean ± SE for every 10 minutes of continuous measurements; n = 5–7. *: significantly different from 10 min within given genotype, P<0.05. #: significantly different from control within given time point, P<0.05.

RER was lower (P<0.05) after 120 min than after 10 min of exercise in both genotypes. Furthermore, IL-6 MKO mice displayed an overall higher (P<0.05) RER than control mice during the 120 min of treadmill exercise (Fig 6).

### Western blots

**Fig 7:**
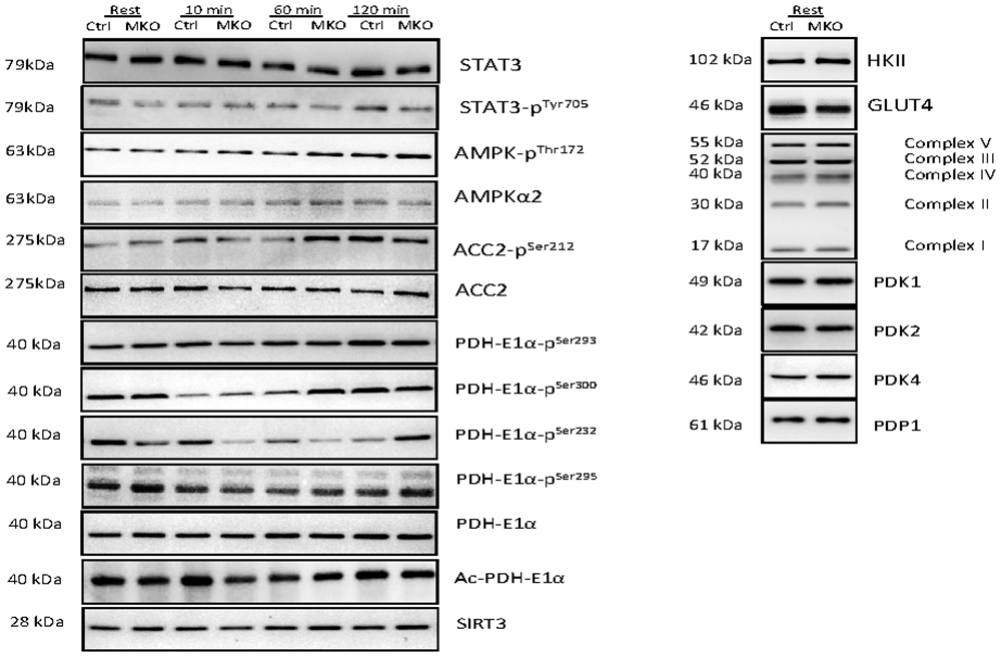
Representative blots of STAT3, STAT3 Tyr705 phosphorylation (phos), AMPK Thr 172 phos, AMPKα2, ACC2 Ser212 phos, ACC2, PDH Ser293 phos, PDH Ser300 phos, PDH Ser232 phos, PDH Ser295 phos, PDH protein, lysine acetylated pdh-E1α protein, sirtuin 3 (SIRT3), Hexokinase II (HKII), GLUT4, OXPHOS complexes I-V, PDK1, PDK2, PDK4 and PDP1 protein content.

For all representative western blots consult Fig 7.

## Discussion

The main findings of the present study are that lack of skeletal muscle IL-6 led to elevated PDHa activity at rest and during exercise without changes in PDHa activity during prolonged exercise as observed in control mice. In addition, IL-6 MKO mice had an overall higher RER during exercise than controls, but maintained the ability to reduce RER during prolonged exercise. Together this indicates that muscle IL-6 influences substrate utilization in skeletal muscle through effects on PDH, but is not required for the shift in substrate use during prolonged exercise.

The observations that the exercise bout in the present study was associated with a gradual decrease in RER and a transient increase in skeletal muscle PDHa activity in control mice as previously reported in humans [7,18,19]), suggest that the experimental setup provides the basis for studying the impact of muscle IL-6 on exercise-induced PDH regulation in skeletal muscle and substrate utilization.

The present findings that IL-6 MKO mice had higher skeletal muscle PDHa activity at rest and after 1h of exercise and did not exhibit a significant change in PDHa activity during exercise show for the first time that lack of skeletal muscle IL-6 affects the regulation of PDH in skeletal muscle. It should be noted that this suggestion is possible although PDHa activity was not significantly different between the genotypes at 2 h of exercise, because the plasma IL-6 levels were increased in both genotypes at 2 h of exercise. Thus, PDHa activity may have been influenced by IL-6 derived from other tissues than skeletal muscle late during exercise. It cannot be excluded that the lack of change in PDHa activity in response to exercise in IL-6 MKO mice is due to PDHa activity being close to full activation already at rest and that a change therefore was not detected during exercise in the present study. However, we have previously observed ~25% higher levels of PDHa activity in mice running at a higher speed and treadmill slope than in the present study (unpublished results). In addition, the observed muscle IL-6 dependent regulation of PDHa activity is in accordance with a previous mouse study showing that a single injection of recombinant IL-6 altered PDHa activity in skeletal muscle, although the change was dependent on the nutritional state as IL-6 lowered PDHa activity in the fed state and increased PDHa activity in the fasted state [31]. Furthermore, the observed impact of muscle-specific IL-6 knockout on PDHa activity suggests that muscle IL-6 normally exerts an inhibitory effect on skeletal muscle PDH with a concomitant decrease in carbohydrate oxidation. Although differences in PDHa activity do not necessarily relate to flux and may therefore not be linked to the difference in RER between IL-6 MKO and control mice, the observed higher RER in IL-6 MKO mice than controls during the exercise bout does support that IL-6 reduces carbohydrate oxidation. On the other hand, the maintained ability of the IL-6 MKO mice to reduce RER during the exercise bout indicates opposite of the hypothesis that muscle IL-6 is not required for a switch towards fat oxidation during prolonged exercise in mice. However, the increase in circulating IL-6 in IL-6 MKO mice is in accordance with a potential effect of non-muscle-derived IL-6 on substrate utilization and hence RER during prolonged exercise. In addition, the higher PDHa activity in IL-6 MKO than control mice not only during exercise, but also at rest emphasizes that muscle IL-6 may not only function as a contraction-induced myokine, but also regulates basal skeletal muscle metabolism.

To elucidate the mechanistic regulatory pattern behind the different regulation of PDHa activity when muscle IL-6 is lacking, the four known PDH phosphorylation sites, site 1: Ser293; site 2: Ser300; site 3: Ser232, and site 4: Ser295, were examined. The observation that phosphorylation of Both sites 2 and 3 followed an overall inverse pattern of the PDHa activity in control mice with robust decreases in absolute phosphorylation during exercise is in accordance with previous findings for site 1 and 2 in humans [19–21]. Moreover, the similar resting and exercise-induced changes in site 2 and 3 and similar lack of change in site 1 and 4 phosphorylation in IL-6 MKO and control mice indicates that diverse regulation of PDH phosphorylation is not the main underlying mechanism for the observed genotype differences in PDHa activity in the present study. In addition, the lack of difference in PDK1, PDK 2, PDK4 and PDP1 protein during exercise in either genotype suggests that protein content does not account for the observed PDH phosphorylation response. Due to the complex site-specific affinity patterns of the various PDKs [43] measuring the activities of all PDK isoforms may likely be needed to fully understand the exercise-induced regulation of PDH phosphorylation and PDHa activity. As it recently has been reported that the mitochondrial NAD+ dependent deacetylase, SIRT3, targets the PDH-E1α subunit in C2C12 cells [22], the acetylation state in skeletal muscle at rest and during exercise might reveal the mechanism explaining the observed changes in PDHa activity with lack of skeletal muscle IL-6. However, the observation that total lysine acetylation of immunoprecipitated PDH-E1α was similar in IL-6 MKO and control mice does not support that acetylation is regulated by skeletal muscle IL-6, although it cannot be excluded that the total acetylation pattern disguises more subtle differences in acetylation that may be more significant to regulation, but this remains to be determined.

As IL-6 has previously been reported to increase AMPK activity both in human [27] and mouse tissue [31,32] and AMPK has been suggested to regulate PDH [33,44], it was hypothesized that AMPK mediates the effects of IL-6 on PDHa activity and substrate utilization. However, the similar exercise-induced AMPK phosphorylation in IL-6 MKO and control mice does not support IL-6 mediated regulation of AMPK as previously reported, although it might be speculated that IL-6-induced regulation of AMPK is overshadowed by stronger effectors in an acute exercise setting.

The finding that plasma glucose at rest was higher in the IL-6 MKO than control mice and the plasma glucose did not drop significantly until the latter stages of the exercise bout might reflect lower glucose removal by skeletal muscle in IL-6 MKO mice or be due to higher hepatic glucose output in IL-6 MKO than in control mice. Furthermore, the observation that basal hexokinase and GLUT4 protein content as well as G-6-P concentrations in skeletal muscle were similar in the two genotypes, while muscle glucose was lower in the IL-6 MKO than control mice may advocate more for a higher glycolytic flux and carbohydrate utilization in mice lacking skeletal muscle IL-6 rather than a lower glucose uptake. In addition, the genotype difference in running endurance during an incremental exercise running test may suggest that the IL-MKO mice were running at a higher relative intensity than the control mice in the current exercise study. However, the IL-6 MKO mice did not require more motivation than the controls during the 2 hour moderate intensity exercise protocol and completed the protocol as the controls in the present study. Furthermore, as a previous human study has reported intensity dependent AMPK regulation [45], the similar exercise-induced AMPK and ACC phosphorylation in the two genotypes in the present study suggests that the relative exercise intensity was equal in the IL-6 MKO and control mice during the exercise bout, which is further supported by the identical decrease in muscle glycogen in the two genotypes during exercise.

The observation that the plasma IL-6 concentrations were significantly elevated after 2 hours of running in control mice is in accordance with previous murine studies [46,47]. The finding that plasma IL-6 concentrations in the control mice reached rather modest levels of ~15 pg/ml points towards the possibility that the loxP inserts in the floxed control mice might affect expression or release of IL-6. Hence, other studies with various treadmill protocols have reported increases in circulating plasma IL-6 to levels between 40–70 pg/ml [30,48]. The observation that skeletal muscle IL-6 mRNA did not increase with the 2 hours of treadmill exercise and even decreased at the end of the exercise bout, opposes previous findings [49], and suggests that exercise did not induce IL-6 transcription in skeletal muscle of the floxed IL-6 mice. It cannot be excluded that exercise may have increased IL-6 mRNA in other muscles thean the investigated quadriceps, but regardless, the skeletal muscle IL-6 mRNA data show that IL-6 was knocked out, which was also confirmed at the level of DNA (S1 Fig). Therefore, the exercise-induced increase in circulating IL-6 levels in the IL-6 MKO mice cannot be originating from skeletal muscle fibers and based on previous findings [50] suggests that IL-6 derived from adipose tissue may be responsible for the increase in plasma IL-6 during exercise in the present experiment. This may indicate that the observed skeletal muscle IL-6 dependent effects on skeletal muscle PDHa activity are mediated by autocrine/paracrine effects of IL-6. However, the identical exercise-induced STAT3 phosphorylation and identical decline in SOCS3 mRNA in the two genotypes suggest that signaling pathways traditionally accredited to be induced by IL-6 were affected similarly by exercise in the two genotypes. Thus, the observed metabolic differences must be exerted through other pathways. In addition, a variety of other cytokines and hormones have been reported to trigger the STAT3 signaling pathway [51,52] and any of these, or non-muscle derived IL-6 may be responsible for the observed STAT3 pathway inductions and explain why this response was also present in IL-6 MKO mice.

The current observation that IL-6 MKO mice displayed a lower exercise capacity than the control mice when submitted to an incremental endurance exercise test is consistent with some [53], but not all previous observations in IL-6 whole body knockout mice [30,54]. The findings that skeletal muscle CS and HAD activities as well as the content of OXPHOS proteins were similar in the two genotypes indicate that skeletal muscle oxidative capacity was unaffected by the lack of skeletal muscle IL-6. Together with the observed higher RER value during exercise in IL-6 MKO mice than controls in the present study this may suggest that the reduced exercise endurance in the IL-6 MKO mice is due to increased carbohydrate/reduced fat utilization relative to controls rather than reduced skeletal muscle oxidative capacity.

In conclusion, the present findings show, for the first time, that lack of skeletal muscle IL-6 leads to elevated PDHa activity in skeletal muscle both at rest and during exercise, and prevented significant changes in PDHa activity during prolonged exercise. Differences in PDH phosphorylation did not seem to explain these genotype differences in PDHa activity and the mechanisms behind are still elusive. In addition, lack of skeletal muscle IL-6 resulted in higher RER during prolonged exercise suggesting that IL-6 normally reduce carbohydrate oxidation during exercise via effects on skeletal muscle PDH.

## Supporting Information

S1 Fig Representative PCR gel of products generated with primers surrounding exon 2 of the IL-6 gene in quadriceps muscle from floxed and IL-6 skeletal muscle-specific knockout mice. A reduction in band size from 1,000 bp to 260 bp in is equal to the loss of exon 2 of the IL-6 gene. WT Ctrl: Wild-type control, Flox Ctrl: Floxed control, MKO Control: Skeletal muscle-specific knockout Control.
(TIF)
