# Supplementary figures and images for: Lack of Skeletal Muscle IL-6 Affects Pyruvate Dehydrogenase Activity at Rest and during Prolonged Exercise
Source: PLoS One. 2016 Jun 21;11(6):e0156460. doi: 10.1371/journal.pone.0156460 (PMC4915712; doi:10.1371/journal.pone.0156460)

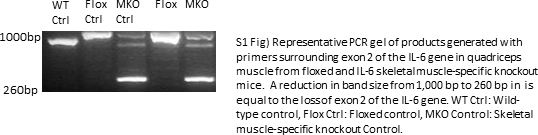

Supplement: S1 Fig — A reduction in band size from 1,000 bp to 260 bp in is equal to the loss of exon 2 of the IL-6 gene. WT Ctrl: Wild-type control, Flox Ctrl: Floxed control, MKO Control: Skeletal muscle-specific knockout Control. (TIF) [file pone.0156460.s001.tif]
